# Health-related quality of life and intensity-specific physical activity in high-risk adults attending a behavior change service within primary care

**DOI:** 10.1371/journal.pone.0226613

**Published:** 2019-12-20

**Authors:** Ellen Eimhjellen Blom, Eivind Aadland, Guri Kaurstad Skrove, Ane Kristiansen Solbraa, Line Merethe Oldervoll

**Affiliations:** 1 Department of Sport, Food and Natural Sciences, Faculty of Education, Arts and Sports, Western Norway University of Applied Sciences, Sogndal, Norway; 2 Department of Public Health and Nursing, Faculty of Medicine and Health Sciences, Norwegian University of Science and Technology, Trondheim, Norway; 3 Department of Social Research, Møreforsking Molde AS, Molde, Norway; Universite Cote d'Azur, FRANCE

## Abstract

**Objectives:**

Health-related quality of life (HRQoL) is an important outcome for health interventions, such as physical activity (PA) promotion among high-risk populations. The aim of this study was to investigate levels of PA and HRQoL, and associations between PA and HRQoL, in participants attending a behavior change service within primary care in Norway.

**Methods:**

Adult participants (≥ 18 years) from 32 Healthy Life Centers (HLCs) in four regions of Norway, who provided valid data on HRQoL (SF-36) and PA (ActiGraph accelerometer) were included (N = 835). HRQoL scores were compared to normative data by independent sample t-tests. Associations between eight dimensions of HRQoL and time spent sedentary (SED), in light PA (LPA) or in moderate to vigorous PA (MVPA) were determined using general linear models adjusted for relevant confounders.

**Results:**

Nineteen percent of the participants (mean age 50; body mass index 32) met PA recommendations of > 150 min MVPA per week. SF-36 scores were 10 to 28 points lower than the norm (all p < 0.001). Positive associations were found between MVPA and the SF-36 dimensions physical functioning, role physical, general health and vitality, (all p < 0.045). LPA was positively associated with physical functioning, role physical, general health, vitality and role emotional (all p < 0.046). Time spent SED was negatively associated with physical functioning, general health, vitality, social functioning and mental health (all p < 0.030).

**Conclusions:**

Individuals attending a Norwegian behavior change service within primary care had low PA level and low HRQoL compared to the general population. Our study suggest there is a positive dose-response relationship between PA and HRQoL, and a negative relationship between SED and HRQoL. Furthermore, that specific PA intensities and SED are related to different dimensions of HRQoL.

## Introduction

Life expectancy has increased substantially worldwide over the last decades [1], resulting in an increased prevalence of people living with chronic diseases and disabilities [2]. Individuals’ self-perceived well-being, function and health status have therefore become important indicators of health policies and health interventions [3]. Health-related quality of life (HRQoL) is a measure that includes several concepts, and covers physical-, mental- and social-health dimensions [3]. Furthermore, as populations’ age and chronic conditions increase, there is a need to promote behaviors that might prevent disability and hospitalization. Physical activity (PA) has well-documented beneficial effects on prevention and treatment of non-communicable diseases (NCDs) and other chronic conditions [4–6] as well as on promotion of HRQoL [7–9]. Nevertheless, the existing knowledge about the relationship between PA and HRQoL is primarily based on self-reported measurements of PA [7, 9], which are known to have low correlation to direct measurements of PA, and to be influenced by several biases, such as recall and social desirability biases [10]. Duration and intensity of PA, in particular, are difficult to recall precisely [10–12], which in turn, hampers the possibility of drawing conclusions about the dose-response relationship between PA and HRQoL.

The current global recommendations for health-enhancing PA for adults and the elderly include PA of minimum moderate intensity (MPA), which equals 3–6 metabolic equivalents (METs), for a total of 150 min per week, or at least 75 min of vigorous intensity PA (VPA) (≥ 6 METs), or any equivalent combination of those, in bouts of at least 10 min duration [13]. Although moderate to vigorous PA (MVPA) is recognized as the most important intensity range to improve physiological health indicators [6], knowledge of relationships between specific PA intensities and HRQoL is sparse [7, 9, 14]. Most previous studies target MVPA only [7, 9]. This practice ignores the possible associations between HRQoL and time spent sedentary (SED) or in light PA (LPA). These behaviors are of special interest in older adults and populations with chronic conditions, who tend to be less physically active than the general population and spend most of their active time in LPA [15–17]. Previous studies investigating associations between HRQoL and SED or LPA among adults and the elderly have reported conflicting results [17–21].

Despite the well-documented health-enhancing effects of PA, there are still many adults and elderly worldwide who are physically inactive (i.e., do not meet PA recommendations). Their numbers represent about 30–90% of adults, depending on assessment methods and study-populations [22–24]. In Norway, only about one-third of the adults meet the PA recommendations when measured objectively [24]. Therefore, broad multi-sector initiatives are needed to promote PA among physically inactive individuals [25]. Thus, as part of the Norwegian national high-risk strategy to achieve the World Health Organization’s (WHO’s) goals of a 25% reduction of premature deaths caused by NCDs, and a 10% reduction in the number of people being physically inactive [25], Healthy Life Centers (HLCs) have been established as a primary health service [26]. HLCs provide help to promote behavior change with regard to PA, diet and smoking. The services target individuals with chronic conditions or at high risk of getting diseases, and who need support to change their behavior (hereafter termed high-risk individuals) [27]. Although 60% of municipalities in Norway have established HLCs [28], knowledge about whom these centers reach is limited. Previous studies of HLCs included small samples and used assessment methods that hamper comparison with the general population’s PA level and HRQoL [29–31]. Large-sampled studies that use objective instruments to assess PA and SED and multi-dimensional instruments to assess HRQoL are lacking.

Therefore, the aims of this study were: a) to explore levels of objectively measured PA and self-reported dimensions of HRQoL among HLC participants and compare the participants’ HRQoL to Norwegian normative data, and b) to study whether time spent in MVPA, LPA and SED was associated with specific dimensions of HRQoL.

## Methods

### Setting and sample

**Fig 1:**
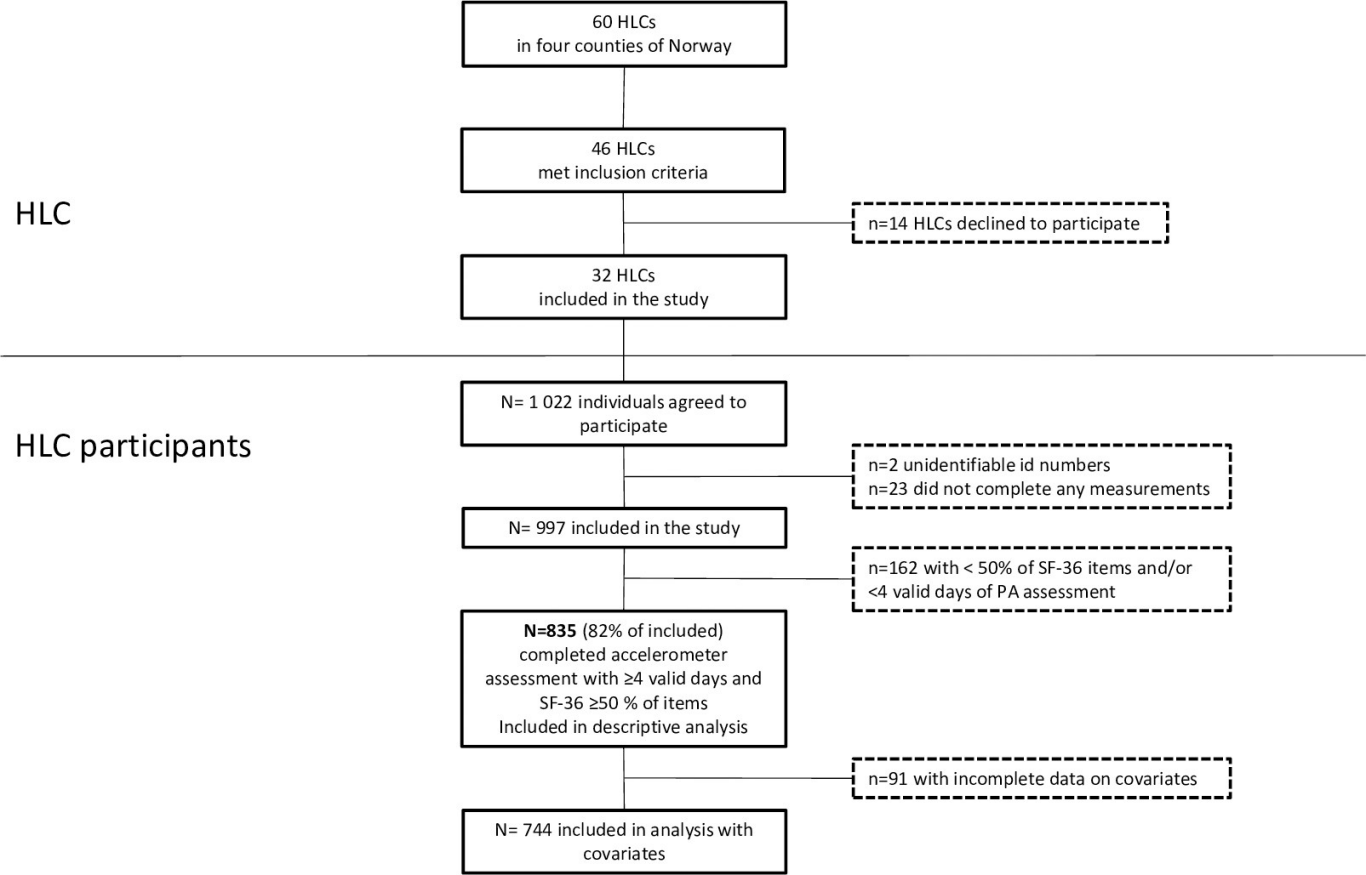
Flow of Healthy Life Centers (HLCs) and study participants.

In the current paper, we present baseline data from a prospective observational study carried out at 32 HLCs in four regions throughout Norway, as shown in Fig 1. The HLCs varied in size, years since establishment and resources available. Individuals 18 years of age or older enrolled at one of the included HLCs were invited to participate in the study. They were self-referred, or referred by general practitioners, other health services, or the Norwegian Labor and Welfare Administration, for guidance to improve PA, diet and/or smoking behaviors. The only exclusion criterion was previous enrollment in a HLC intervention during the last 6 months. Data collection was performed in the period between August 2016 and February 2018. Study design and inclusion procedures are explained in more detail elsewhere [32]. Among those initially eligible to participate, about 33% declined to take part, 10% were not invited because the professional judgement of HLC staff was that they would be unable to complete questionnaires and physical tests (e.g. due to serious cognitive, physical or mental impairments), and 1% had begun their intervention before agreeing to participate in the study and were therefore excluded. A total of 1,022 individuals agreed to participate and provided written informed consent, representing 56% of those eligible to participate. The estimation of individuals taking part in the study was based on reports from 26 of the 32 HLCs. In the current study, we included participants with valid assessments of PA and HRQoL, as described in Fig 1. The study was approved by the Regional Committee for Medical and Health Research Ethics (ref. 2016/546/REK midt).

Norwegian reference values of HRQoL from the survey by Jacobsen et al. [33] (N = 2,118, aged 18–80 years with a mean age 55.7 years, conducted in 2015, were used to compare the study population’s HRQoL to the Norwegian norms [33].

### Variables

#### Health-related quality of life

HRQoL was assessed by the generic, multidimensional questionnaire Medical Outcome Study 36-item short form (SF-36) version 1.0. [34, 35]. SF-36 is translated and validated in the Norwegian population, and updated reference values have been recently published [33, 36]. The instrument consists of 36 items that assess the following eight dimensions: physical functioning (10 items), role limitations due to physical problems (role physical) (four items), bodily pain (two items), general health perceptions (general health) (five items), vitality (four items), social functioning (two items), role limitations due to emotional problems (role emotional) (three items), mental health generally (five items) and one item measuring health transition (not used in the current study) [35]. Participants who completed 50% or more of each dimension’s items were included. Imputation of missing data and transformation of item scores were incorporated according to the standard SF-36 algorithm [35]. Missing values were replaced with the person’s mean score of completed items within the same dimension. Each dimension gave a raw score by averaging the items of the corresponding dimension, which were linearly transformed to a scale of zero (lowest health state) to 100 (highest health state) [35].

#### Physical activity level and sedentary time

PA level and SED were assessed by ActiGraph GT3X accelerometer (ActiGraph, LLC, Pensacola, Florida, USA). ActiGraph is the most widely used accelerometer and correlates well with energy expenditure derived from the doubly labeled water technique [37]. The accelerometer measures acceleration in three axes providing a value called counts. The number of counts per minute (cpm) over a given period provides information about activity, intensity and duration. Despite its limitations in measuring upper-body movement, cycling, water activities, and incline walking with heavy loads, it is valid and reliable for measuring daily PA and time spent in different intensities and SED [10, 38]. Accelerometers were sent by mail to the participants together with information about how to use it and a reminder poster. The participants were instructed to put on the accelerometer by placing it on the right hip the morning after they received it, and were told to wear it for seven consecutive days during waking hours, except when taking a shower or bath. After 7 days, they were asked to deliver the monitor to HLC staff, who returned it together with questionnaires in a prepaid envelope.

Data processing criteria according to Migueles and colleagues were used [39], which are the same used in Norwegian population surveys [24, 40]. Accelerometers were initialized in ActiLife software (v 6.13.3, ActiGraph) with a sample frequency of 30 Hz, sleeping mode enabled, and were programmed to start monitoring 2 days after they were sent out by mail, with no defined stop time. Data was downloaded using the same software, normal filtering option and a 10-second epoch setting. Files were later reintegrated to 60-second epochs to match the Norwegian surveys’ data processing [24]. Non-wear time was defined as a minimum of 60 consecutive minutes with zero counts with allowance of up to 2 min of non-zero counts. We inspected all files individually to identify and remove noise from mail delivery. Wear criteria for a valid measurement was at least 600 min per day for at least 4 days. Only the vertical axis was used to enable application of the well-established cut points from Troiano et al. [41], as also previously used in Norwegian population samples [24]: SED (0–99 cpm), LPA (100–2,019 cpm), MPA (2,020–5,998 cpm), VPA (≥ 5,999 cpm), and MVPA (≥ 2,020 cpm) intensities. PA outcomes were intensity-specific PA levels reported as average time spent in MVPA, VPA, MPA, LPA and SED per day (min/day), and time spent in 10-min bouts of MVPA (≥ 2,020 cpm) allowing for 2 min drop time (min/day). Overall PA level was reported as total PA (cpm) and average steps per day. Meeting the current recommendations for PA was defined as having an average daily sum of 10-min MVPA bouts that was ≥ 21.4 min/day (150 min per week) [13, 24, 42]. Average wear time of the accelerometer per day (wear time (min/day)) was calculated as total wear time on valid days divided by number of valid days.

#### Covariates

Based on previous findings of associations with HRQoL [43, 44], and after stepwise analyses, we included the following self-reported covariates: gender; age (continuous); and level of education reported as primary school max 10 years, high school max 13 years, college/university ≤ 3 years, and college/university > 3 years. Marital status was reported as married/partner/cohabitant, divorced/separated/terminated partnership/cohabitation, widow/widower, and never been married/partner/cohabitant, dichotomized as married/partner or not married/partner. Smoking status was reported as never smoked, stopped smoking, smoking sometimes, and currently smoking daily, dichotomized as smoking or not smoking. Chronic conditions were self-reported through the following pre-defined categories: no disease, overweight/obese, muscle/skeletal, hypertension, mental problems, mental disease, diabetes, cardiovascular disease (CVD), lung/respiratory, cancer (current or former), and others (with space to specify). Number of chronic conditions reported were summed and categorized as none, one, or multimorbidity of two, three, or ≥ four chronic conditions (continuous). Body mass index (BMI) was calculated as kg/m^2^ based on measured height and body mass assessed by HLC staff, and categorized according to WHO’s classification (Underweight, BMI < 20; Normal, BMI 20–24; Overweight, BMI 25–29; Obese, BMI ≥ 30) [45]. The continuous variable of BMI was used as covariate in analysis of associations, whereas the categories were used for stratification. Occupational status was registered through interviews by HLC staff, but excluded from analysis due to collinearity with chronic conditions.

### Statistical analysis

Categorical data are presented as frequencies and continuous variables as means and standard deviations (SDs). Descriptive results of PA level, SED and HRQoL variables are presented as unadjusted means (SD) by gender and age (< 65 / ≥ 65 years). Sensitivity analyses were performed as independent sample t-tests for continuous variables and Mann Whitney U tests for categorical variables between participants (n = 835) and nonparticipants (n = 162). Differences in HRQoL dimension scores between HLC participants and the general Norwegian population are presented as mean scores (SD) and mean differences, and independent sample t-tests were performed to explore statistical differences between populations. According to previous work, 0.2, 0.5 and 0.8 times the SD is considered as respectively small, moderate and large differences in HRQoL scores. A moderate difference is generally considered as a clinically relevant and meaningful difference [46, 47].

Linear relationships between MVPA, LPA or SED and HRQoL dimensions were investigated using scatter plots and by calculation of the p-value for a linear trend with MVPA, LPA and SED as continuous independent variables (S1 Table). Because of several nonlinear relationships, individuals were classified into quartiles of time spent in MVPA, LPA and SED. HRQoL dimension scores are presented as unadjusted means (SD) by the quartiles of MVPA, LPA and SED, and analysis of variance (ANOVA) were performed to test the overall differences in scores between the groups (p-values). The quartiles were as follows for MVPA: low: 0–8 min [reference]; mid-low: 8–16.9 min; mid-high:17–31 min; and high: > 31.5 min. LPA: low: < 211 min [reference]; mid-low: 211–259 min; mid-high: 260–317 min; and high: ≥ 318 min. SED: low: < 496 min [reference]; mid-low: 497–562 min; mid-high: 563–618 min; and high: > 618 min.

Univariate general linear models (GLMs) were performed to investigate associations between each of the eight dimensions of HRQoL as outcomes and each quartile of MVPA, LPA and SED as exposures (independent variables). MVPA, LPA and SED were included in separate models for each of the eight HRQoL dimensions because of the collinearity among PA- and SED variables. However, we also performed sensitivity analyses including adjustment for SED in models for MVPA and adjustment for MVPA in models for LPA and SED. All models were adjusted for the following covariates: age, gender, educational level, marital status, smoking status, chronic condition, BMI and accelerometer wear time. Interaction analyses were performed by including possible interaction terms into all of the GLMs. Gender, age, BMI and chronic conditions were tested as potential moderators, and all four interaction terms (i.e. gender*MVPA, age*MVPA, BMI*MVPA and chronic conditions*MVPA etc.) were included simultaneously in each of the models. Because of unconcise interaction effects across the HRQoL dimensions, associations between MVPA, LPA or SED with the eight HRQoL dimensions are reported as main effect estimates (B coefficients) along with 95% confidence intervals (Cis) for the total sample. The B coefficients represent differences in SF-36 score points (0–100 scale) compared to the reference quartile. Based on previous work by Sloan et al., 3%, 8% and 13% of the HRQoL scoring range might be considered as small, moderate and large differences, respectively [46]. Hence, B ≥ 8 might be considered as a clinically meaningful difference, which corresponds to 0.5 times the SD [46]. Interactions are reported with p-values, and for the significant interactions, stratified analyses are presented.

All tests were two-sided, with probability values of 0.05 indicating statistically significant findings. Statistical analyses were performed using IBM Statistics for Windows, Version 24.0 and 25.0 (SPSS Inc., Chicago, Illinois, USA).

## Results

### Participant characteristics

In total, 835 participants (73% women) provided valid data on PA and SF-36, mean age 49.6 years, ranging from 18–87 years. Ninety-one percent were of Norwegian origin. Thirty percent reported having one chronic condition, whereas 29%, 22%, and 11% reported multimorbidity of two, three, and four or more conditions, respectively. The participants’ mean BMI was 32.4 (7.4) kg/m^2^. Detailed information about participants’ characteristics is presented in Table 1. Sensitivity analyses showed that the included individuals were older than the individuals who had been excluded from analysis (mean 50 vs. 42 years, respectively, p < 0.001). However, the two groups did not differ in terms of gender, level of education or BMI.

**Table 1:** Participant characteristics.

Variables	% (n)
**Educational level n = 832**	
** Primary school, 0–10 years**	17.8 (148)
** High school, 11–13 years**	49.4 (411)
** College/university, ≤ 3 years**	19.4 (161)
** College/university, > 3 years**	13.5 (112)
**Occupational status n = 831**	
** Disability pension (full-time)**	31.6 (264)
** Working (not sick leave or disability pension)**	25.9 (216)
** Part-time working and sick leave/ disability pension**	15.4 (128)
** Retiree**	15.4 (128)
** Sick leave (full-time)**	8.3 (69)
** Student**	1.8 (15)
**Other^a^**	5.1 (42)
**Chronic conditions^b^ n = 828**	
** No disease**	10.0 (83)
** Overweight/obese**	49.0 (406)
** Muscle/skeletal**	41.4 (343)
** Hypertension**	27.4 (227)
** Mental problems**	15.6 (129)
** Mental disease**	11.6 (96)
** Diabetes**	11.6 (96)
** CVD**	10.0 (83)
** Lung/respiratory**	10.0 (83)
** Cancer, current or former**	3.5 (29)
**Other^c^**	16.4 (136)
**Weight classification n = 796**	
** Underweight (BMI < 20)**	0.5 (4)
** Normal (BMI 20–24)**	13.2 (105)
** Overweight (BMI 25–29)**	25.0 (199)
** Obese (BMI ≥ 30)**	61.4 (488)
**Smoking status n = 825**	
** Never smoked**	37.2 (307)
** Stopped smoking**	39.5 (326)
** Smoking sometimes**	5.0 (41)
** Smoking daily**	18.3 (151)
**Marital status n = 835**	
** Married/partner**	64.1 (535)
** Not married/partner**	35.9 (300)

^a^Maternity leave, homemakers, i.a.

^b^It was possible to report more than one condition, therefore the total percentage adds up to more than 100%.

^c^Other chronic conditions include fatigue/ME, headache, hypothyroidism, rheumatic diseases, neurologic, psoriasis, syndromes, hypercholesterolemia, allergy, i.a.

CVD = Cardiovascular diseases, BMI = Body mass Index

### Physical activity level

The included participants had a median of seven valid days of PA registration. Overall, 19% of the participants achieved the recommended amount of PA (≥ 150 min/week in 10-min bouts of MVPA). The participants’ intensity-specific PA levels, average wear time per day and overall PA level are shown in Table 2, by gender and age categories.

**Table 2:** Physical activity level presented as mean (SD) by gender and age categories.

	Total	Women	Men	Adults	Older adults
PA variable	N = 835	n = 606	n = 229	n = 688*	n = 141*
**MVPA (min/day)**	22.7 (19.1)	22.3 (18.2)	23.8 (21.3)	24.1 (19.4)	16.4 (16.2)
**Vigorous (min/day)**	0.4 (2.0)	0.5 (2.2)	0.4 (1.2)	0.5 (2.2)	0.1 (0.3)
**Moderate (min/day)**	22.4 (18.7)	22.0 (17.7)	23.5 (21.0)	23.7 (19.0)	16.4 (16.1)
**Light (min/day)**	269 (80)	282 (76)	235 (79)	275 (80)	239 (72)
**Sedentary (min/day)**	562 (93)	548 (89)	598 (94)	556 (92)	591 (92)
**Time in 10-min bouts of MVPA (min/day)**	10.9 (14.9)	10.8 (14.4)	11.2 (16.1)	11.5 (15.3)	8.4 (12.4)
**Wear time (min/day)**	853 (81)	852 (80)	857 (83)	855 (82)	846 (76)
**Total PA (cpm)**	265 (116)	271 (111)	248 (127)	276 (115)	211 (106)
**Steps per day**	5,878 (2,570)	6,009 (2,444)	5,530 (2,851)	6,135 (2,548)	4,680 (2,341)
**Meeting PA recommendations (%)**	18.6	18.6	18.3	19.6	13.5

PA = physical activity, MVPA = moderate to vigorous intensity PA, cpm = counts per minute.

Adults (18–64 years), Older adults (65–87 years)

*n = 6 did not report age.

### Health-related quality of life

Among the included sample (n = 835) with > 50% completed items within all SF-36 dimensions, 93.5% competed all items, 5.1% missed one item and 1.3% missed 2–4 items overall. For detailed information on missing item pattern, see S2 Table.

The HLC participants’ HRQoL scores were 10 to 28 points lower than the general Norwegian population’s scores across all dimensions and genders (all p-values < 0.001, and all differences above the clinically meaningful difference of 8 points). Table 3 shows HLC participants’ HRQoL scores compared to reference values from the general Norwegian adult population [33] along with differences in mean scores between the two groups.

**Table 3:** HRQoL scores (0–100) (SD) for Healthy Life Centre (HLC) population compared to the normal population (norm) by gender.

	Women			Men		
HRQoL dimensions	HLC scores (SD)	Norm scores (SD)	Difference (p)	HLC scores (SD)	Norm scores (SD)	Difference (p)
Physical functioning	74.8 (17.6)	84.9 (21.0)	-10.1 (p<0.001)	75.8 (19.0)	88.1 (17.0)	-12.3 (p<0.001)
Role physical	46.4 (41.9)	72.6 (39.6)	-26.2 (p<0.001)	50.9 (41.6)	78.9 (35.3)	-28.0 (p<0.001)
Bodily pain	52.2 (27.4)	66.9 (26.5)	-14.7 (p<0.001)	58.1 (29.8)	72.1 (25.4)	-14.0 (p<0.001)
General health	52.7 (21.9)	72.6 (22.5)	-19.9 (p<0.001)	51.0 (21.4)	73.4 (20.8)	-22.4 (p<0.001)
Vitality	40.0 (19.9)	57.2 (20.6)	-17.2 (p<0.001)	45.1 (20.0)	61.9 (18.9)	-16.8 (p<0.001)
Social functioning	67.8 (28.3)	85.7 (21.6)	-17.9 (p<0.001)	72.2 (28.0)	89.0 (19.3)	-16.8 (p<0.001)
Role emotional	64.2 (42.8)	87.4 (28.6)	-23.2 (p<0.001)	64.1 (40.9)	89.5 (26.3)	-25.4 (p<0.001)
Mental health	67.4 (19.7)	79.9 (14.8)	-12.5 (p<0.001)	71.4 (20.5)	81.9 (13.8)	-10.5 (p<0.001)

Differences in scores between the HLC- and norm populations, with corresponding p-values, are presented for each dimension.

HRQoL scores varied across quartile levels of MVPA, LPA and SED (Table 4). For MVPA, significant differences between quartiles were found for physical functioning, role physical and general health. For LPA, we observed significant differences between quartiles for physical functioning, role physical, general health, vitality, social functioning and role emotional. For SED, significant differences between quartiles were found for physical functioning and mental health.

**Table 4:** HRQoL dimension scores (0–100, (SD)) by quartile levels of MVPA, LPA and SED.

	Physical functioning	Role physical	Bodily pain	General health	Vitality	Social functioning	Role emotional	Mental health
**MVPA**								
Low	65.4 (19.9)	37.2 (40.2)	50.0 (28.6)	49.0 (21.4)	41.2 (19.1)	69.1 (28.1)	63.4 (42.6)	71.0 (19.4)
Mid-low	75.7 (16.8)	47.9 (41.0)	53.3 (28.1)	50.9 (21.5)	40.1 (20.4)	68.1 (29.0)	65.2 (40.9)	66.6 (21.1)
Mid-high	78.7 (16.8)	51.7 (43.3)	55.4 (28.6)	53.7 (22.2)	42.3 (21.3)	70.7 (28.5)	66.1 (42.2)	67.9 (19.8)
High	79.9 (14.7)	52.9 (41.4)	56.3 (27.2)	55.1 (21.5)	42.0 (19.3)	68.1 (27.7)	62.0 (43.5)	68.6 (19.4)
p-value	< 0.001**	< 0.001**	0.104	0.018*	0.680	0.760	0.753	0.160
**LPA**								
Low	70.0 (20.5)	39.5 (41.1)	53.0 (28.1)	46.8 (20.9)	38.1 (18.8)	67.7 (29.0)	55.3 (43.9)	67.1 (20.9)
Mid-low	73.4 (18.5)	51.8 (42.3)	56.3 (29.6)	52.0 (22.0)	41.6 (20.3)	67.3 (28.6)	64.5 (42.1)	69.1 (20.6)
Mid-high	76.9 (15.8)	44.3 (40.8)	51.1 (26.7)	52.4 (21.8)	41.0 (20.5)	67.3 (29.0)	63.5 (42.0)	66.7 (20.4)
High	79.5 (15.6)	54.4 (42.0)	54.9 (28.3)	57.2 (21.2)	44.6 (20.0)	73.7 (26.1)	72.9 (39.6)	71.0 (17.6)
p-value	< 0.001**	0.001**	0.252	< 0.001**	0.013*	0.049*	0.001**	0.087
**SED**								
Low	76.5 (16.5)	49.7 (42.4)	53.2 (28.9)	52.2 (22.9)	43.6 (19.8)	69.7 (28.9)	63.9 (43.3	68.0 (19.1)
Mid-low	76.2 (17.5)	43.1 (41.1)	51.8 (27.7)	50.3 (21.4)	39.3 (20.7)	65.8 (28.1)	60.5 (42.3	64.6 (20.0)
Mid-high	75.6 (17.6)	49.0 (41.7)	55.2 (28.0)	54.0 (20.8)	41.0 (20.9)	68.8 (27.9)	65.1 (42.0	69.8 (20.3)
High	72.1 (19.8)	48.7 (42.2)	54.9 (28.3)	52.4 (21.8)	41.8 (18.6)	71.7 (28.1)	67.1 (41.6	71.5 (20.0)
p-value	0.045*	0.348	0.574	0.372	0.175	0.198	0.433	0.003**

HRQoL = Health-related quality of life, MVPA = Moderate- to vigorous physical activity, LPA = Light physical activity, SED = Sedentary time

HRQoL scores are presented as unadjusted means (SD). Overall differences between quartile levels of MVPA, LPA, and SED respectively are presented as p-values, *p < 0.05. **p < 0.01

Ranges and medians for the MVPA-. LPA- and SED quartiles are as follows

MVPA Low: 0–8 min (median 4 min), Mid-low: 8–16.9 min (median 12 min), Mid-high: 17–31 min (median 24 min), High: > 31 min (median 46 min).

LPA Low: < 211 min (median 178 min), Mid-low: 211–259 min (median 239 min), Mid-high: 260–317 min (median 287 min), High: ≥ 318 min (median 365min).

SED Low: < 496 min (median 455 min), Mid-low: 497–562 min (median 531 min), Mid-high: 563–618 min (median 587 min), High: > 618 min (median 666 min).

### Relationship between HRQoL and MVPA, LPA and SED

In total 774 participants provided valid data on all of the covariates in addition to PA- and HRQoL variables. Table 4 shows the associations between each of the eight HRQoL dimensions and MVPA, LPA and SED expressed in quartiles of time spent in each of the PA intensities or SED adjusted for covariates. Time spent in MVPA was positively associated with the dimensions physical functioning, role physical, general health and vitality. For physical functioning, role physical and general health, we observed an increase in score with increased MVPA, where individuals in the lower quartile (median 4 min/day) had significantly lower scores than all the other quartiles in physical functioning and lower scores than the mid-high and high quartiles in role physical and general health. For vitality, there was a nonlinear relationship with MVPA, and only the mid-high quartile had a significantly higher score than the individuals in the lower quartile. Clinically meaningful estimates (B ≥ 8 points) were found for physical functioning (low vs. the other quartiles) and role physical (low vs. mid-high and high quartiles). See Table 5 for detailed information.

**Table 5:** Associations between HRQoL dimensions (outcomes) and MVPA-, LPA- or SED (exposures).

	Physical functioning	Role physical	Bodily pain	General health	Vitality	Social functioning	Role emotional	Mental health
**MVPA**								
Low (reference)								
Mid-low	7.8 (4.4, 11.2)**	5.3 (-3.3, 13.8)	2.4 (-3.3, 8.1)	3.2 (-0.9, 7.2)	2.5 (-1.4, 6.4)	1.9 (-3.6, 7.5)	4.5 (-4.0, 12.9)	-1.9 (-5.7, 1.8)
Mid-high	9.6 (6.1, 13.0)**	9.5 (0.8, 18.1)*	4.5 (-1.3, 10.3)	5.7 (1.6, 9.8)**	4.0 (0.1, 8.0)*	3.7 (-2.0, 9.3)	4.8 (-3.7, 13.4)	-0.7 (-4.5, 3.1)
High	10.7 (7.2, 14.2)**	9.9 (1.3, 18.6)*	4.6 (-1.2, 10.4)	7.2 (3.1, 11.3)**	3.7 (-0.3, 7.7)	1.2 (-4.4, 6.9)	1.1 (-7.4, 9.6)	-0.9 (-4.7, 2.9)
**LPA**								
Low (reference)								
Mid-low	2.4 (-1.1, 5.8)	10.9 (2.4, 19.4)*	3.8 (-1.8, 9.5)	4.8 (0.8, 8.8)*	5.2 (1.4, 9.0)**	0.5 (-5.0, 6.0)	7.9 (-0.4, 16.2)	2.2 (-1.5, 5.9)
Mid-high	4.7 (1.0, 8.3)*	2.1 (-6.7, 11.0)	-1.1 (-7.0, 4.8)	5.2 (0.9, 9.4)*	6.0 (2.0, 10.0)**	0.5 (-5.3, 6.2)	7.2 (-1.5, 15.9)	-0.5 (-4.4, 3.4)
High	5.3 (1.5, 9.1)**	9.5 (0.2, 18.8)*	0.9 (-5.3, 7.1)	7.8 (3.4, 12.3)**	9.5 (5.3, 13.8)**	5.7 (-0.4, 11.8)	14.4 (5.2, 23.5)**	3.3 (-0.8, 7.3)
**SED**								
Low (reference)								
Mid-low	-0.5 (-4.0, 2.9)	-5.9 (-14.4, 2.6)	-0.2 (-5.9, 5.5)	-1.2 (-5.3, 2.8)	-4.6 (-8.4,-0.7)*	-6.1 (-11.6, -0.6)*	-6.3 (-14.6, 2.1)	-5.2 (-8.9, -1.5)**
Mid-high	-2.1 (-5.6, 1.5)	-2.3 (-11.0, 6.4)	1.5 (-4.2, 7.3)	-1.3 (-5.4, 2.8)	-6.0 (-9.9, -2.0)**	-6.3 (-11.9, -0.7)*	-7.0 (-15.5, 1.5)	-2.3 (-6.1, 1.5)
High	-7.3 (-11.3, -3.4)**	-6.8 (-16.4, 2.8)	-1.3 (-7.7, 5.1)	-5.6 (-10.2, -1.0)*	-8.1 (-12.5, -3.8)**	-7.1 (-13.4, -0.9)*	-7.2 (-16.6, 2.3)	-2.8 (-7.0, 1.4)

HRQoL = Health-related quality of life, MVPA = Moderate- to vigorous physical activity, LPA = Light physical activity, SED = Sedentary time

Associations between outcomes and exposures were controlled for gender, age, BMI, accelerometer wear time, educational level, chronic conditions, smoking status and marital status, presented as main effect estimates (B coefficients (95% CI)). Significant associations at *p < 0.05, **p < 0.01

Time spent in LPA was positively associated with the dimensions physical functioning, role physical, general health, vitality and role emotional, with a linear relationship between LPA and all of the dimensions except for role physical. Individuals in the lower quartile (median 2.9 hours LPA/day) had significantly lower scores in the general health and vitality dimensions than those in the three other quartiles. For physical functioning, there was a significant difference in score between the lower quartile and the mid-high and high LPA quartiles. However, for role physical, there was a nonlinear relationship, and for role emotional only individuals in the upper quartile had a significantly higher score than those in the lower quartile. Clinically meaningful estimates (B ≥ 8 points), were found for role physical (low vs. mid-low and high quartiles), general health (low vs. high quartile), vitality (low vs. high quartile) and role emotional (low vs. high quartile). For detailed information, see Table 5.

Time spent SED was negatively associated with physical functioning, general health, vitality, social functioning and mental health (Table 5). For vitality and social functioning there was a linear relationship with decreased scores with increasing time spent SED, and all other quartiles had significantly lower scores than the low reference quartile (median 7.6 hours). For physical functioning and general health, only the higher quartile (median 11 hours SED/day) had significantly lower scores than the low reference quartile. For mental health, there was a nonlinear relationship, in which only individuals in the mid-low group (median 8.8 hours SED/day) had significantly lower scores than the low reference quartile. Clinically meaningful estimates (B ≥ 8 points), were found for vitality (low vs. high quartile).

When models for SED and LPA were mutually adjusted for MVPA in sensitivity analysis, the following associations became non-significant: between MVPA and vitality (all p > 0.235), between LPA and physical functioning (all p > 0.104), and between SED and physical functioning and general health (all p > 0.057).

We observed significant interaction effects (p < 0.05) between age and MVPA, LPA and/or SED on physical functioning, role physical and mental health (S3 Table). Furthermore, there were significant interactions between BMI and MVPA, LPA and/or SED on physical functioning, bodily pain and social functioning. Gender and chronic health conditions interacted with SED only on general health. See S3 Table for detailed information.

Table 6 shows associations for physical functioning, role physical and mental health stratified on age. The associations between MVPA and LPA and the physical dimensions were strongest in the older age groups (> 50 years), and no significant relationships were observed in the youngest age group (18–34 years). For mental health, the associations with LPA (positive association) and SED (negative association) were strongest in the youngest age group and no significant relationships were observed in the oldest age group (65–87 years) (Table 6).

**Table 6:** Associations between physical functioning, role physical or mental health as outcomes and MVPA/LPA/SED as exposures stratified by age groups.

	18–34 (n = 137)	35–49 (n = 216)	50–64 (n = 291)	65–87 (n = 130)
**Physical functioning**				
**MVPA (ref low)**				
Mid-low	6.6 (-3.5, 16.7)	2.9 (-3.8, 9.6)	4.5 (-1.1, 10.0)	17.0 (8.7, 25.4)**
Mid-high	5.5 (-4.6, 15.7)	4.0 (-2.5, 10.6)	8.8 (3.0, 14.7)**	17.9 (10.4, 25.3)**
High	-1.4 (-11.6, 8.8)	9.4 (2.5, 16.2)**	10.5 (5.0, 15.9)**	21.0 (13.0, 29.1)**
**LPA (ref low)**				
Mid-low	-6.0 (-12.3, 7.5)	-2.3 (-9.6, 5.0)	7.7 (1.8, 13.5)**	5.3 (-2.5, 13.2)
Mid-high	-3.1 (-12.7, 6.4)	0.6 (-6.4, 7.7)	8.3 (2.2, 14.5)**	9.8 (0.6, 18.9)*
High	-2.4 (-12.3, 7.5)	-0.2 (-7.8, 7.5)	11.6 (5.4, 17.8)**	7.3 (-2.9, 17.6)
**Role physical**				
**LPA (ref low)**				
**Mid-low**	8.7 (-10.2, 27.6)	-3.9 (-21.8, 14.0)	10.8 (-4.6, 26.2)	22.8 (5.9, 39.8)**
**Mid-high**	6.8 (-13.7, 27.3)	-11.9 (-29.2, 5.3)	-1.2 (-17.4, 15.0)	24.0 (4.2, 43.8)*
**High**	9.9 (-11.3, 31.1)	5.1 (-13.5, 23.8)	1.3 (-14.9, 17.6)	37.0 (14.9, 59.2)**
**Mental health**				
**LPA (ref low)**				
Mid-low	7.0 (-2.2, 16.2)	3.1 (-5.7, 12.0)	-0.8 (-6.9, 5.2)	2.3 (-4.4, 8.9)
Mid-high	12.7 (2.7, 22.7)*	-3.2 (-11.8, 5.3)	-7.3 (-13.7, -0.9)*	1.2 (-6.5, 9.0)
High	16.3 (6.0, 26.7)**	4.8 (-4.4, 14.0)	-3.0 (-9.5, 3.4)	-3.4 (-12.0, 5.3)
**SED (ref low)**				
Mid-low	-8.9 (-17.5, -0.4)*	-6.0 (-13.6, 1.6)	-2.4 (-8.2, 3.4)	-1.9 (-11.5, 7.6)
Mid-high	-4.1 (-13.7, 5.6)	-4.0 (-11.8, 3.8)	0.1 (-5.8, 6.0)	1.7 (-7.1, 10.4)
High	-17.6 (-27.7, -7.5)**	-7.6 (-17.4, 2.1)	6.9 (0.4, 13.4)*	1.8 (-7.4, 11.1)

Associations are presented as coefficients (B) (95% CI), controlled for gender, BMI, accelerometer wear time, educational level, chronic conditions, smoking status and marital status. Significant associations at *p < 0.05, **p < 0.01.

Stratification by age groups were based on significant interaction effects between age and the presented PA-intensities and/or SED on these dimensions.

Table 7 presents associations for bodily pain and social functioning stratified on BMI. The associations between PA or SED and bodily pain were only sporadic. However, for social functioning we observed that the only weight category with significant associations was the obese group (BMI ≥ 30).

**Table 7:** Associations between bodily pain and social functioning as outcomes and MVPA/LPA /SED as exposures stratified by BMI categories.

	BMI < 25 (n = 104)	BMI 25–29.9 (n = 194)	BMI ≥ 30 (n = 474)
**Bodily pain**			
**MVPA (ref low)**			
Mid-low	5.3 (-6.5, 27.3)	-1.3 (-13.1, 10.5)	1.2 (-1.6, 13.1)
Mid-high	4.5 (-12.7, 21.7)	3.3 (-8.6, 15.1)	4.1 (-3.2, 11.5)
High	10.4 (-13.0, 23.6)	-3.5 (-15.5, 8.5)	5.7 (-6.0, 8.4)
**LPA (ref low)**			
Mid-low	-9.0 (-27.0, 9.0)	2.3 (-9.3, 14.0)	6.4 (1.0, 17.5)*
Mid-high	-10.0 (-26.1, 6.1)	-5.9 (-18.0, 6.2)	3.3 (-4.2, 10.9)
High	-11.2 (-27.9, 5.5)	-7.1 (-19.3, 5.1)	9.2 (-0.7, 13.4)
**SED (ref low)**			
Mid-low	9.3 (-6.5, 25.1)	6.5 (-7.6, 17.1)	-6.1 (-13.6, 1.3)
Mid-high	-6.8 (-22.9, 9.3)	12.5 (0.7, 24.2)*	-3.9 (-11.3, 3.5)
High	11.9 (-5.0, 28.9)	4.8 (-4.4, 17.5)	-9.2 (-17.8, -0.6)*
**Social functioning**			
**MVPA (ref low)**			
Mid-low	-6.1 (-23.5, 11.3)	-2.4 (-20.0, 6.1)	4.4 (-2.3, 11.1)
Mid-high	-5.4 (-21.8, 11.0)	0.8 (-12.1, 13.7)	6.7 (-0.2, 13.6)
High	-6.1 (-25.8, 6.4)	-6.9 (-15.3, 10.4)	7.0 (0.2, 13.9)*
**LPA (ref low)**			
Mid-low	-3.8 (-21.1, 13.5)	-3.3 (-16.0, 9.5)	1.6 (-5.0, 8.2)
Mid-high	-3.7 (-19.1, 11.8)	-4.5 (-17.8, 8.7)	2.7 (-4.3, 9.8)
High	-2.7 (-18.8, 13.4)	2.9 (-10.4, 16.2)	9.6 (1.9, 17.3)*
**SED (ref low)**			
Mid-low	-4.6 (-19.9, 10.6)	-2.8 (-14.9, 9.2)	-8.7 (-15.6, -1.7)*
Mid-high	-12.9 (-28.5, 2.6)	1.5 (-11.4, 14.3)	-10.0 (-16.9, -3.1)**
High	-1.3 (-17.7, 15.1)	-1.9 (-15.5, 11.7)	-11.7 (-19.7, -3.7)**

Associations are presented as coefficients (B) (95% CI), controlled for gender, age, accelerometer wear time, educational level, chronic conditions, smoking status and marital status. Significant associations at *p < 0.05, **p < 0.01. Stratification by BMI categories were based on significant interaction effects between BMI and MVPA, LPA and SED for these dimensions.

## Discussion

The main results from this study show that the individuals recruited to Norwegian HLCs had a low physical activity level and reported significantly lower HRQoL in all dimensions compared to data from the general Norwegian population. A positive relationship between HRQoL and both moderate to vigorous- (MVPA) and light physical activity (LPA), and a negative relationship with time spent sedentary (SED) was observed. The different PA intensities (MVPA and LPA) and SED were clinically meaningful associated (B coefficients ≥ 8) with different dimensions of HRQoL. MVPA was related to physical functioning and role physical, LPA to role physical, general health, vitality and role emotional, while SED was related to vitality. However, the associations for some of the dimensions were dependent on age and BMI category.

The Norwegian HLCs target high-risk individuals with low PA level [27]. Previous studies of Norwegian HLC’s have found that the HLCs recruited individuals with high PA level compared to the general Norwegian population [29, 31]. Contradictory to these findings, the present study suggest that the HLCs reach physically inactive individuals. Furthermore, the vast majority were obese, had low level of education, were on sick leave or social benefits and had a high degree of multimorbidity. Our results indicating that the HLCs reach a high-risk group is essential, as previous studies have found that behavior change counseling within primary care is effective in improving health and increasing PA level when reaching high-risk individuals [48, 49], contradictory to conflicting results when targeting a healthy population without any risk factors [50]. The conflicting results in terms of HLC participants’ PA level could possibly be attributed to smaller samples in the previous studies, or that they used self-reported PA [31], a different accelerometer device, or different data processing methods to classify the participants’ PA levels [29]. PA level and SED in the current study was measured with the same device, and by using the same data processing criteria, as the Norwegian surveillance system for PA [24]. Adults in the present study accumulated on average 24 min of MPA per day compared to 38 and 36 min (men and women, respectively) in the general Norwegian population [24]. Older adults in the Norwegian population accumulated 33 min (male) and 30 min (female) of MPA, while the elderly HLC participants engaged in 16 min of MPA daily [24]. Only 20% of adults and 14% of older adults in the HLC sample met current PA recommendations, compared to 33% in the general adult population and 31% among older adults in Norway [24]. However, our findings are in line with results for obese individuals in the general population, where 16% met the PA guidelines [24, 51].

The HRQoL scores in the present study population ranged from 40 in the dimension vitality (women) to 76 in physical functioning (men). We observed substantially lower scores in all dimensions compared to the general population, ranging from 26 and 28 points lower in the role physical dimension (women and men, respectively) to 11 and 13 points lower in the mental health dimension (men and women, respectively) [33]. Previous studies have considered an 8-point difference in SF-36 scores as clinically important [46], which is comparable to the estimate of 0.5 SD [52]. Thus, the high-risk population in our study reported substantially, and clinically meaningful lower scores than the general Norwegian population across all dimensions [33]. These results confirm previous findings of lower HRQoL among individuals with chronic conditions compared to generally healthy populations [53, 54].

Furthermore, we found that HRQoL scores varied between groups with different PA- or SED levels. Existing evidence about the relationship between PA and HRQoL in adults and the elderly is mainly based on self-reported PA data [7, 9], which has limitations concerning measuring the duration of activities of certain intensities or sedentary time precisely. The use of accelerometers enabled us to classify individuals’ PA and SED levels more precisely than self-reported data does, and hence, investigate the dose-response relationship between HRQoL and the various PA intensity levels. After adjustment for confounders, we observed a positive gradient of HRQoL scores in the dimensions physical functioning, role physical and general health across levels of MVPA. These results are in line with previous findings in cross-sectional studies [55, 56]. However, in the present study, the differences in scores between the two highest levels of MVPA was minor (only about one point). Our findings support previous studies suggesting there might be a threshold at which excessive MVPA do not have any further positive relationship with HRQoL [18, 57, 58]. Furthermore, we found that the level of MVPA needed to score clinically meaningful higher than the individuals in the lower quartile did, differed between the various dimensions. For physical functioning this level was median 12 min MVPA per day, while for role physical this level was 24 min MVPA per day. For general health, the differences in scores between levels of MVPA were less than 8 points, and hence not considered as clinical relevant.

For LPA, only individuals in the higher quartile, spending a median of 6 hours LPA per day, reported clinically meaningful higher scores than the lower quartile in the dimensions general health, vitality and role emotional. For the dimension role physical there was a non-linear trend where both the mid-low (median 4 hours LPA per day) and the higher quartile reported higher scores compared to the lower quartile. Vitality was the only dimension where there was observed a clinically relevant difference between levels of SED, in which individuals spending a median 11 hours SED per day had lower scores compared to the least sedentary individuals (median 8 hours SED per day). These findings are in line with previous studies indicating that PA at different intensities and SED are associated with different dimensions of HRQoL [17, 20], and furthermore that the dose-response relationship might differ between dimensions.

Most previous studies have investigated the relationship between HRQoL and PA of only moderate-to vigorous intensity [7, 9]. Our study confirms previous consistent findings of positive associations between MVPA and the physical-related dimensions (physical functioning and role physical) [7, 9]. However, we found that MVPA was not associated with any of the mental-related dimensions (role emotional, mental health, social functioning, or vitality) [34], in which previous evidence seems to be contradictory [7, 9, 18]. On the other hand, we found that LPA was associated with mental-related dimensions (vitality and role emotional) [34]. The conflicting results regarding MVPA’s association to mental-related dimensions might partly be attributed to the variety of thresholds at which PA is defined as moderate- to vigorous, based on the wide specter of measurement instruments used [7, 9]. Questionnaires are prone to recall bias, and especially duration of every-day activities, which not are experienced as breath taking, may be hard to recall precisely, while one tend to over-report activity of moderate- and hard intensity [10]. This might have led to misclassification of LPA as MVPA in previous studies using self-reported PA. However, the current study found no associations between neither MVPA nor LPA and SED and the specific mental health dimension. Though, when we investigated the relationship stratified by age, we found statistical and clinically relevant associations between mental health and both LPA and SED for individuals in the youngest age group (18 to 34 years), indicating that the relationship possibly could be dependent on age. Furthermore, about one third of the current study’s population reported mental disorders. Individuals with mental disorders have shown to score significantly lower than the general population in mental-related dimensions [54]. Hence, the association between mental-related dimensions and PA might have been attenuated by the individuals’ chronic conditions, as we only controlled for number of chronic conditions, and not for specific disorders. Further studies are therefore needed to explore the relationship between mental health and PA in populations with multimorbidity.

The current study found no significant associations between any of the PA intensities and bodily pain or social functioning, which is in line with previous findings in the reviews by Pucci et al. [9] and Bize et al. [7]. However, we found that BMI had an interaction effect with PA and SED on bodily pain and social functioning, which might indicate that these dimensions are influenced by moderating factors such as BMI. As the current study is based on cross-sectional data, we are limited in interpreting any causality. Thus, it possible that higher BMI has an impact on bodily pain and social functioning, which in turn might influence the choice of activities, or to not engage in physical activity. Further studies with longitudinal designs are needed to investigate the causality of the relationships.

### Strengths and limitations

One of the strengths of the current study is the relatively large sample of high-risk individuals from a wide range of HLCs in four regions in Norway. Thus, we believe this is a more representative sample of Norwegian HLC participants compared to the smaller samples included in previous studies [29–31]. Other important strengths are the adjustments for several confounders in the association analysis, the use of objective PA assessments, and a widely used, multidimensional generic HRQoL instrument, both being the same measurement methods used in previous large population surveys [24, 33]. This strengthens comparisons between populations. Although objective measurements of PA generally have higher validity than subjective assessments, there are some limitations with the use of accelerometers. First, the accelerometer is not able to capture type or characteristics of the activity performed. Setting or purpose of the activity might play an important role in terms of emotions related to the activity and hence the activity’s impact on quality of life. Furthermore, cut-points used to define intensity-specific PA are not uniformly defined [39], which adds complexity to making comparisons across studies. The cross-sectional design of this study limits the interpretation of the causal direction of the association between PA and HRQoL, as an individual’s physical or mental health could have an impact on his or her physical activity level.

In conclusion, the results from the present study showed that the majority of participants attending HLCs had low PA level and relatively low HRQoL compared to the general population. Furthermore, we found a positive relationship between PA and HRQoL and a negative relationship between SED and HRQoL. These relationships varied according to the intensity of PA and differed across dimensions of HRQoL, age, and BMI groups. Our findings might contribute to the understanding of the dose-response relationship between PA of specific intensities or SED and the various dimensions of HRQoL. However, longitudinal studies are needed to further investigate possible causal relationships.

## Supporting information

S1 Table Linear trends between MVPA, LPA or SED and HRQoL dimensions. Linear trends between exposures (MVPA, LPA and SED) and HRQoL dimensions (outcomes) are presented as p-values.
(PDF)

S2 Table Missing item pattern. Frequency (%) and number (n) of individuals missing items in each of the eight health-related quality of life (HRQoL) dimensions.
(PDF)

S3 Table Interaction effects. Interactions between MVPA, LPA, SED and possible moderators on HRQoL dimensions.
(PDF)

S1 File Source dataset. (SAV)

## References

[1] GBD 2017 Mortality Collaborators. Global, regional, and national age-sex-specific mortality and life expectancy, 1950–2017: a systematic analysis for the Global Burden of Disease Study 2017. Lancet. 2018;392(10159):1684–735. 10.1016/S0140-6736(18)31891-9 30496102 PMC6227504

[2] GBD 2017 Disease and injury Incidence and Prevalence Collaborators. Global, regional, and national incidence, prevalence, and years lived with disability for 354 diseases and injuries for 195 countries and territories, 1990–2017: a systematic analysis for the Global Burden of Disease Study 2017. Lancet. 2018;392(10159):1789–858. 10.1016/S0140-6736(18)32279-7 30496104 PMC6227754

[3] Fayers PM, Machin D. Quality of life: the assessment, analysis, and reporting of patient-reported outcomes 3rd ed Chichester, West Sussex, England: Wiley Blackwell; 2016. 651 p.

[4] GBD Risk Factor Collaborators. Global, regional, and national comparative risk assessment of 84 behavioural, environmental and occupational, and metabolic risks or clusters of risks for 195 countries and territories, 1990–2017: a systematic analysis for the Global Burden of Disease Study 2017. Lancet. 2018;392(10159):1923–94. 10.1016/S0140-6736(18)32225-6 30496105 PMC6227755

[5] Pedersen BK, Saltin B. Exercise as medicine—evidence for prescribing exercise as therapy in 26 different chronic diseases. Scand J Med Sci Sports. 2015;25 Suppl 3:1–72. 10.1111/sms.12581 26606383

[6] Physical Activity Guidelines Advisory Committee. 2018 Physical Activity Guidelines Advisory Committee Scientific Report. Washington, DC: U.S. Department of Health and Human Services; 2018

[7] Bize R, Johnson JA, Plotnikoff RC. Physical activity level and health-related quality of life in the general adult population: A systematic review. Prev Med. 2007;45(6):401–15. 10.1016/j.ypmed.2007.07.017 17707498

[8] Rejeski WJ, Mihalko SL. Physical activity and quality of life in older adults. J Gerontol A Biol Sci Med Sci. 2001;56 Spec No 2:23–35. 11730235 10.1093/gerona/56.suppl_2.23

[9] Pucci GC, Rech CR, Fermino RC, Reis RS. Association between physical activity and quality of life in adults. Rev Saude Publica. 2012;46(1):166–79. 10.1590/s0034-89102012000100021 22249758

[10] Warren JM, Ekelund U, Besson H, Mezzani A, Geladas N, Vanhees L. Assessment of physical activity—a review of methodologies with reference to epidemiological research: a report of the exercise physiology section of the European Association of Cardiovascular Prevention and Rehabilitation. Eur J Cardiovasc Prev Rehabil. 2010;17(2):127–39. 10.1097/HJR.0b013e32832ed875 20215971

[11] Sallis JF, Saelens BE. Assessment of physical activity by self-report: status, limitations, and future directions. Res Q Exerc Sport. 2000;71 Suppl 2:1–14. 10.1080/02701367.2000.11082780 25680007

[12] Prince SA, Adamo KB, Hamel ME, Hardt J, Connor Gorber S, Tremblay M. A comparison of direct versus self-report measures for assessing physical activity in adults: a systematic review. Int J Behav Nutr Phys Act. 2008;5:56 10.1186/1479-5868-5-56 18990237 PMC2588639

[13] World Health Organization. Global recommendations on physical activity for health. Geneva, Switzerland; 2010 26180873

[14] Bertheussen GF, Romundstad PR, Landmark T, Kaasa S, Dale O, Helbostad JL. Associations between physical activity and physical and mental health -a HUNT 3 study. Med Sci Sports Exerc. 2011;43(7):1220–8. 10.1249/MSS.0b013e318206c66e 21131869

[15] Ashe MC, Miller WC, Eng JJ, Noreau L. Older adults, chronic disease and leisure-time physical activity. Gerontology. 2009;55(1):64–72. 10.1159/000141518 18566534 PMC3167824

[16] Keats MR, Cui Y, DeClercq V, Dummer TJB, Forbes C, Grandy SA, et al Multimorbidity in Atlantic Canada and association with low levels of physical activity. Prev Med. 2017;105:326–31. 10.1016/j.ypmed.2017.10.013 28987335

[17] Buman MP, Hekler EB, Haskell WL, Pruitt L, Conway TL, Cain KL, et al Objective light-intensity physical activity associations with rated health in older adults. Am J Epidemiol. 2010;172(10):1155–65. 10.1093/aje/kwq249 20843864 PMC3004766

[18] Kim J, Im JS, Choi YH. Objectively measured sedentary behavior and moderate-to-vigorous physical activity on the health-related quality of life in US adults: The National Health and Nutrition Examination Survey 2003–2006. Qual Life Res. 2017;26(5):1315–26. 10.1007/s11136-016-1451-y 27837382

[19] Fox KR, Stathi A, McKenna J, Davis MG. Physical activity and mental well-being in older people participating in the Better Ageing Project. Eur J Appl Physiol. 2007;100(5):591–602. 10.1007/s00421-007-0392-0 17285318

[20] Ku PW, Fox KR, Liao Y, Sun WJ, Chen LJ. Prospective associations of objectively assessed physical activity at different intensities with subjective well-being in older adults. Qual Life Res. 2016;25(11):2909–19. 10.1007/s11136-016-1309-3 27153854

[21] Loprinzi PD. Joint associations of objectively-measured sedentary behavior and physical activity with health-related quality of life. Prev Med Rep. 2015;2:959–61. 10.1016/j.pmedr.2015.11.004 26844174 PMC4721439

[22] Guthold R, Stevens GA, Riley LM, Bull FC. Worldwide trends in insufficient physical activity from 2001 to 2016: a pooled analysis of 358 population-based surveys with 1.9 million participants. Lancet Glob Health. 2018;6(10):e1077–e86. 10.1016/S2214-109X(18)30357-7 30193830

[23] Tucker JM, Welk GJ, Beyler NK. Physical Activity in U.S. Adults: Compliance with the Physical Activity Guidelines for Americans. Am J Prev Med. 2011;40(4):454–61. 10.1016/j.amepre.2010.12.016 21406280

[24] Hansen BH, Kolle E, Steene-Johannessen J, Dalene KE, Ekelund U, Anderssen SA. Monitoring population levels of physical activity and sedentary time in Norway across the lifespan. Scand J Med Sci Sports. 2019;29(1):105–12. 10.1111/sms.13314 30276928

[25] World Health Organization. Global action plan for the prevention and control of noncommunicable diseases 2013–2020. Geneva: WHO; 2013

[26] Helse- og omsorgsdepartementet. NCD-strategi 2013–2017. For forebygging, diagnostisering, behandling og rehabilitering av fire ikke-smittsomme folkesykdommer; hjerte- og karsykdommer, diabetes, kols og kreft [NCD-Strategy 2013–2017. For the prevention, diagnosis, treatment and rehabilitation of four noncommunicable diseases: cardiovascular disease, diabetes, COPD and cancer]. Oslo: Helse- og omsorgsdepartementet [Norwegian Ministery of Health and Care Services]; 2013–2017.

[27] Helsedirektoratet. Veileder for kommunale frisklivssentraler Etablering, organisering og tilbud [Recommendations for municipal healthy life centres]. Oslo: Helsedirektoratet [The Norwegian Directorate of Health]; 2016

[28] Ekornrud T, Thonstad M. Frisklivssentralar i kommunane. Ei kartlegging og analyse av førebyggande og helsefremjande arbeid og tilbod i norske kommunar i perioden 2013–2016 [Healthy Life centres in the municipalities A survey and analysis of health-promoting work and offers in Norwegian municipalities during the period 2013–2016]. Oslo—Kongsvinger: Statistics Norway; 2018

[29] Samdal GB, Meland E, Eide GE, Berntsen S, Abildsnes E, Stea TH, et al Participants at Norwegian Healthy Life Centres: Who are they, why do they attend and how are they motivated? A cross-sectional study. Scand J Public Health. 2018:1403494818756081. 10.1177/1403494818756081 29516790

[30] Lerdal A, Celius EH, Pedersen G. Prescribed exercise: a prospective study of health-related quality of life and physical fitness among participants in an officially sponsored municipal physical training program. Journal Of Physical Activity & Health. 2013;10(7):1016–23. 23136380 10.1123/jpah.10.7.1016

[31] Følling IS, Kulseng B, Midthjell K, Rangul V, Helvik A-S. Individuals at high risk for type 2 diabetes invited to a lifestyle program: characteristics of participants versus non-participants (the HUNT Study) and 24-month follow-up of participants (the VEND-RISK Study). BMJ Open Diabetes Res Care. 2017;5(1). 10.1136/bmjdrc-2016-000368 PMC5574427 28878932

[32] Blom EE, Oldervoll L, Aadland E, Solbraa AK, Skrove GK. Impact and implementation of Healthy Life Centres, a primary-care service intervention for behaviour change in Norway: Study design. Scand J Public Health. 2019:1403494819856832 10.1177/1403494819856832 31213167

[33] Jacobsen EL, Bye A, Aass N, Fossa SD, Grotmol KS, Kaasa S, et al Norwegian reference values for the Short-Form Health Survey 36: development over time. Qual Life Res. 2018;27(5):1201–12. 10.1007/s11136-017-1684-4 28808829 PMC5891566

[34] Ware JE Jr. SF-36 health survey update. Spine (Phila Pa 1976). 2000;25(24):3130–9. 11124729 10.1097/00007632-200012150-00008

[35] Ware JE, Snow KK, Kosinski M, Gandek B. SF-36 health survey Manual and interpretation guide. Boston, Massachusets: The Health Institute, New England Medical Center Hospital; 1993.

[36] Jacobsen EL, Bye A, Aass N, Fossa SD, Grotmol KS, Kaasa S, et al Correction to: Norwegian reference values for the Short-Form Health Survey 36: development over time. Qual Life Res. 2018;27(5):1213–5. 10.1007/s11136-017-1708-0 29168123 PMC5891560

[37] Plasqui G, Westerterp KR. Physical activity assessment with accelerometers: an evaluation against doubly labeled water. Obesity (Silver Spring). 2007;15(10):2371–9. 17925461 10.1038/oby.2007.281

[38] Aadland E, Ylvisåker E. Reliability of the Actigraph GT3X+ Accelerometer in Adults under Free-Living Conditions. PLOS ONE. 2015;10(8):e0134606 10.1371/journal.pone.0134606 26274586 PMC4537282

[39] Migueles JH, Cadenas-Sanchez C, Ekelund U, Delisle Nystrom C, Mora-Gonzalez J, Lof M, et al Accelerometer Data Collection and Processing Criteria to Assess Physical Activity and Other Outcomes: A Systematic Review and Practical Considerations. Sports Med. 2017;47(9):1821–45. 10.1007/s40279-017-0716-0 28303543 PMC6231536

[40] Hansen BH, Kolle E, Dyrstad SM, Holme I, Anderssen SA. Accelerometer-determined physical activity in adults and older people. Med Sci Sports Exerc. 2012;44(2):266–72. 10.1249/MSS.0b013e31822cb354 21796052

[41] Troiano RP, Berrigan D, Dodd KW, Masse LC, Tilert T, McDowell M. Physical activity in the United States measured by accelerometer. Med Sci Sports Exerc. 2008;40(1):181–8. 10.1249/mss.0b013e31815a51b3 18091006

[42] Helsedirektoratet. Anbefalinger om kosthold, ernæring og fysisk aktivitet [Recommendations on diet, nutrition and physical activity]. Oslo: Helsedirektoratet [The Norwegian Directorate of Health]; 2014

[43] Riise T, Moen BE, Nortvedt MW. Occupation, lifestyle factors and health-related quality of life: the Hordaland Health Study. J Occup Environ Med. 2003;45(3):324–32. 10.1097/01.jom.0000052965.43131.c3 12661190

[44] Wolin KY, Glynn RJ, Colditz GA, Lee IM, Kawachi I. Long-term physical activity patterns and health-related quality of life in U.S. women. Am J Prev Med. 2007;32(6):490–9. 10.1016/j.amepre.2007.02.014 17533064 PMC1950448

[45] World Health Organization. Obesity: preventing and managing the global epidemic: report of a WHO Counsultation. Geneva, Switzerland: WHO; 2000 11234459

[46] Sloan J, Symonds T, Vargas-Chanes D, Fridley B. Practical Guidelines for Assessing the Clinical Significance of Health-Related Quality of Life Changes within Clinical Trials. Drug Information Journal. 2003;37(1):23–31.

[47] Norman GR, Sloan JA, Wyrwich KW. The truly remarkable universality of half a standard deviation: confirmation through another look. Expert Rev Pharmacoecon Outcomes Res. 2004;4(5):581–5. 10.1586/14737167.4.5.581 19807551

[48] LeFevre ML. Behavioral counseling to promote a healthful diet and physical activity for cardiovascular disease prevention in adults with cardiovascular risk factors: U.S. Preventive Services Task Force Recommendation Statement. Ann Intern Med. 2014;161(8):587–93. 10.7326/M14-1796 25155419

[49] Campbell F, Holmes M, Everson-Hock E, Davis S, Buckley Woods H, Anokye N, et al A systematic review and economic evaluation of exercise referral schemes in primary care: a short report. Health Technol Assess. 2015;19(60):1–110. 10.3310/hta19600 26222987 PMC4781341

[50] Grossman DC, Bibbins-Domingo K, Curry SJ, Barry MJ, Davidson KW, Doubeni CA, et al Behavioral Counseling to Promote a Healthful Diet and Physical Activity for Cardiovascular Disease Prevention in Adults Without Cardiovascular Risk Factors: US Preventive Services Task Force Recommendation Statement. JAMA. 2017;318(2):167–74. 10.1001/jama.2017.7171 28697260

[51] Hansen BH, Anderssen SA, Steene-Johannessen J, Ekelund U, Nilsen AK, Andersen ID, et al Fysisk aktivitet og sedat tid blant voksne og eldre i Norge—Nasjonal kartlegging 2014–2015 [Physical activity and sedentary time among adults and the elderly in Norway—National survey 2014–2015]. Oslo: Helsedirektoratet [The Norwegian Directorate of Health]; 2015

[52] Norman GR, Sloan JA, Wyrwich KW. Interpretation of changes in health-related quality of life: the remarkable universality of half a standard deviation. Med Care. 2003;41(5):582–92. 10.1097/01.MLR.0000062554.74615.4C 12719681

[53] Alonso J, Ferrer M, Gandek B, Ware JE Jr., Aaronson NK, Mosconi P, et al Health-related quality of life associated with chronic conditions in eight countries: results from the International Quality of Life Assessment (IQOLA) Project. Qual Life Res. 2004;13(2):283–98. 10.1023/b:qure.0000018472.46236.05 15085901

[54] Wang HM, Beyer M, Gensichen J, Gerlach FM. Health-related quality of life among general practice patients with differing chronic diseases in Germany: cross sectional survey. BMC Public Health. 2008;8:246 10.1186/1471-2458-8-246 18638419 PMC2515099

[55] Vuillemin A, Boini S, Bertrais S, Tessier S, Oppert J-M, Hercberg S, et al Leisure time physical activity and health-related quality of life. Prev Med. 2005;41(2):562–9. 10.1016/j.ypmed.2005.01.006 15917053

[56] Wendel-Vos GCW, Schuit AJ, Tijhuis MAR, Kromhout D. Leisure Time Physical Activity and Health-Related Quality of Life: Cross-Sectional and Longitudinal Associations. Qual Life Res. 2004;13(3):667–77. 10.1023/B:QURE.0000021313.51397.33 15130029

[57] Brown DW, Brown DR, Heath GW, Balluz L, Giles WH, Ford ES, et al Associations between physical activity dose and health-related quality of life. Med Sci Sports Exerc. 2004;36(5):890–6. 10.1249/01.mss.0000126778.77049.76 15126726

[58] Balboa-Castillo T, Leon-Munoz LM, Graciani A, Rodriguez-Artalejo F, Guallar-Castillon P. Longitudinal association of physical activity and sedentary behavior during leisure time with health-related quality of life in community-dwelling older adults. Health Qual Life Outcomes. 2011;9:47 10.1186/1477-7525-9-47 21708011 PMC3142200

