# Supplementary material for: Health-related quality of life and intensity-specific physical activity in high-risk adults attending a behavior change service within primary care
Source: PLoS One. 2019 Dec 20;14(12):e0226613. doi: 10.1371/journal.pone.0226613 (PMC6924667; doi:10.1371/journal.pone.0226613)
Supplement: S1 Table — Linear trends between exposures (MVPA, LPA and SED) and HRQoL dimensions (outcomes) are presented as p-values. (PDF) [file pone.0226613.s001.pdf]

S1 Table. Linear trends between MVPA, LPA or SED and HRQoL dimensions.

Exposure<br>Outcome	**MVPA**	**LPA**	**SED**
**Physical functioning**	<.001*	<.001*	.001*
**Role physical**	<.001*	<.001*	.312
**Bodily pain**	.034*	.232	.521
**General health**	<.001*	<.001*	.383
**Vitality**	.338	.001*	.643
**Social functioning**	.811	.001*	.169
**Role emotional**	.321	<.001*	.775
**Mental health**	.522	.008*	.018*
**Role physical**	<.001*	<.001*	.312

Linear trends between exposures (MVPA, LPA and SED) and HRQoL dimensions (outcomes) presented as p-values. *p< 0.05 represents that the relationship fits a linear trend.
