# Supplementary material for: Health-related quality of life and intensity-specific physical activity in high-risk adults attending a behavior change service within primary care
Source: PLoS One. 2019 Dec 20;14(12):e0226613. doi: 10.1371/journal.pone.0226613 (PMC6924667; doi:10.1371/journal.pone.0226613)
Supplement: S2 Table — Frequency (%) and number (n) of individuals missing items in each of the eight health-related quality of life (HRQoL) dimensions. (PDF) [file pone.0226613.s002.pdf]

S2 Table. **Missing item pattern**

**HRQoL Dimension**	**Number of missing items**	**% (n)**
Physical functioning	1	1.3 (11)
	2	0 (0)
	3	0.1 (1)
Role physical	1	0.7 (6)
	2	0.2 (2)
Bodily pain	1	0.1 (1)
General health	1	1.2 (10)
	2	0.1 (1)
Vitality	1	0.7 (6)
Social functioning	1	1.6 (13)
Role emotional	1	0.6 (5)
Mental health	1	0.8 (7)

Frequency (%) and number (n) of individuals missing items in each of the eight health-related quality of life (HRQoL) dimensions
