# Supplementary material for: Health-related quality of life and intensity-specific physical activity in high-risk adults attending a behavior change service within primary care
Source: PLoS One. 2019 Dec 20;14(12):e0226613. doi: 10.1371/journal.pone.0226613 (PMC6924667; doi:10.1371/journal.pone.0226613)
Supplement: S3 Table — Interactions between MVPA, LPA, SED and possible moderators on HRQoL dimensions. (PDF) [file pone.0226613.s003.pdf]

S3 Table. Interaction effects. Interactions between MVPA, LPA, SED and possible moderators on HRQoL dimensions**.** N=774

	**Physical functioning**	**Role physical**	**Bodily pain**	**General health**	**Vitality**	**Social functioning**	**Role emotional**	**Mental health**
**Interaction terms**								
**MVPA**								
Gender* MVPA	P=.471	P=.414	P=.922	P=.534	P=.317	P=.393	P=.534	P=.940
Age* MVPA	P<.001*	P=.274	P=.202	P=.177	P=.522	P=.956	P=.051	P=.643
BMI* MVPA	P=.404	P=.713	P=.024*	P=.505	P=.187	P=.004*	P=.344	P=.178
Chronic conditions * MVPA	P=.201	P=.849	P=.223	P=.155	P=.512	P=.876	P=.530	P=.455
**LPA**								
Gender*LPA	P= .063	P=.308	P=.986	P=.965	P=.386	P=.729	P=.650	P=.667
Age*LPA	P=.007*	P=.013*	P=.680	P=.626	P=.771	P=.817	P=.799	P=.006*
BMI*LPA	P=.993	P=.240	P<.000*	P=.246	P=.064	P=.016*	P=.534	P=.492
Chronic conditions*LPA	P=.168	P=.725	P=.978	P=.183	P=.392	P=.580	P=.098	P=.616
**SED**								
Gender* SED	P=.137	P=.622	P=.973	P=.038*	P=.677	P=.185	P=.848	P=.629
Age *SED	P=.072	P=.227	P=.479	P=.551	P=.108	P=.137	P=.840	P=.002*
BMI* SED	P=.129	P=.381	P=.004*	P=.371	P=.715	P=.012*	P=.716	P=.968
Chronic conditions*SED	P=.324	P=.385	P=.673	P=.006**	P=.944	P=.975	P=.798	P=.860

*P value < .05 indicates a significant interaction. MVPA: moderate- to vigorous physical activity, LPA: light physical activity, SED: sedentary time, HRQoL: health-related quality of life, BMI: Body mass index
